# Low Intensity Focused Ultrasound for Non-invasive and Reversible Deep Brain Neuromodulation—A Paradigm Shift in Psychiatric Research

**DOI:** 10.3389/fpsyt.2022.825802

**Published:** 2022-02-24

**Authors:** Amanda R. Arulpragasam, Mascha van 't Wout-Frank, Jennifer Barredo, Christiana R. Faucher, Benjamin D. Greenberg, Noah S. Philip

**Affiliations:** ^1^VA RR&D Center for Neurorestoration and Neurotechnology, VA Providence Healthcare System, Providence, RI, United States; ^2^Department of Psychiatry and Human Behavior, Alpert Medical School of Brown University, Providence, RI, United States; ^3^COBRE Center for Neuromodulation, Butler Hospital, Providence, RI, United States

**Keywords:** non-invasive brain stimulation, acoustic stimulation, low intensity focused ultrasound, neuromodulation, brain stimulation

## Abstract

This article describes an emerging non-invasive neuromodulatory technology, called low intensity focused ultrasound (LIFU). This technology is potentially paradigm shifting as it can deliver non-invasive and reversible deep brain neuromodulation through acoustic sonication, at millimeter precision. Low intensity focused ultrasound's spatial precision, yet non-invasive nature sets it apart from current technologies, such as transcranial magnetic or electrical stimulation and deep brain stimulation. Additionally, its reversible effects allow for the causal study of deep brain regions implicated in psychiatric illness. Studies to date have demonstrated that LIFU can safely modulate human brain activity at cortical and subcortical levels. Due to its novelty, most researchers and clinicians are not aware of the potential applications and promise of this technique, underscoring the need for foundational papers to introduce the community to LIFU. This mini-review and synthesis of recent advances examines several key papers on LIFU administered to humans, describes the population under study, parameters used, and relevant findings that may guide future research. We conclude with a concise overview of some of the more pressing questions to date, considerations when interpreting new data from an emerging field, and highlight the opportunities and challenges in this exciting new area of study.

## Introduction

Non-invasive brain stimulation is a rapidly growing area of psychiatric research and clinical practice. As our understanding of the neurocircuitry underlying psychiatric disorders has grown, so too has the development of technologies to modulate disease-relevant brain targets. Though promising, most non-invasive stimulation approaches lack the spatial precision of other invasive techniques, limiting their therapeutic utility.

The most widely used non-invasive brain stimulation modalities are transcranial magnetic simulation (TMS) and transcranial electrical stimulation. Transcranial magnetic simulation has garnered considerable attention following its success in treating pharmacoresistant depression (1, 2) and obsessive compulsive disorder (3), and evidence in treating other psychiatric illnesses (4–7). Transcranial magnetic simulation uses alternating magnetic fields to induce electrical current in the brain. Traditional TMS designs suffer from diffuse induced electric fields that decay exponentially as a function of cortical depth (8). The large extent of TMS-induced neuronal activation limits this technique's ability to directly target and engage deeper brain regions and circuits involved in psychopathology, leading to a reliance on indirect polysynaptic “downstream” modulation from cortical targeting. These limitations have motivated the development of “deep” TMS coils, such as the Hesed (H) and double cone coils, to target deeper brain targets. These two coils have demonstrated efficacy in the treatment of depression (9). While they can directly stimulate targets at depths of up to 4 cm, they still have reduced spatial focality compared to figure-of-eight TMS coils (8, 10). Further, the intensity required to depolarize deeper targets (>4 cm) exceeds the upper limit in current rTMS safety guidelines (i.e., risk of seizure) (10).

Transcranial electrical stimulation also has promise in psychiatric research and treatment (11–13). This technique delivers electrical current between scalp electrodes to produce weak electrical fields in the brain. Several variations of this method exist (e.g., direct currents, alternating currents, or random noise), but all result in a diffuse electric field that is difficult to restrict to a specific brain target (14, 15), thus limiting precise effects. Therefore, both transcranial magnetic and electrical stimulation indirectly target and engage regions involved in psychiatric illnesses, yet lack the spatial precision used in invasive approaches, such as deep brain stimulation.

Low intensity focused ultrasound (LIFU) holds great promise as a novel approach to brain stimulation (16). Unlike transcranial magnetic and electrical stimulation, LIFU can directly modulate activity within deep brain structures with high spatial precision (17), and the effects of even brief sonication may last several hours (18). Low intensity focused ultrasound applies acoustic energy to reversibly modulate neural activity (19). By yielding reversible effects with spatial focality, LIFU distinguishes itself from high intensity focused ultrasound, which is used to thermally ablate tissue in the treatment of Parkinson's and tremor [reviewed in Bachu et al. (20)], as well as transcranial ultrasound, which provides non-focal and reversible sonication. In this mini-review, we describe this emerging and promising new technology, highlighting key papers and other efforts that describe safety and potential efficacy in humans, as well as current limitations and future considerations.

## Ultrasound Neuromodulation

**Figure 1:**
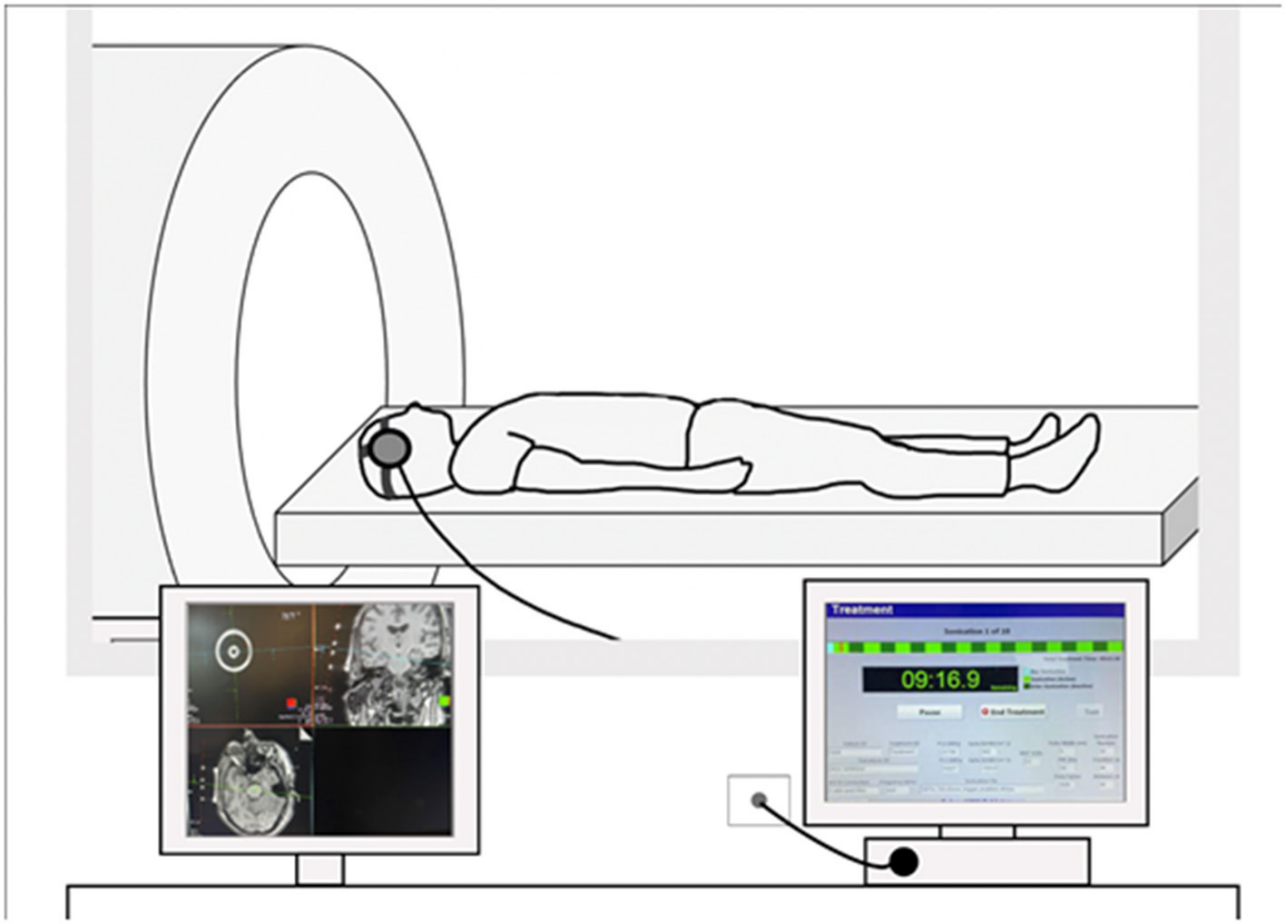
Example of an MRI-guided LIFU setup. Transducer element is affixed to the head using a head strap and connected to a console. (Left) Example of MRI-guided targeting on a structural MRI scan. Transducers include built-in fiducials for targeting via the Siemens MRI interface. (Right) Example image of the sonication console where light green represents active sonication and dark green represents off-line time.

Ultrasound is defined as a sound or acoustic wave higher than 20 kHz (i.e., above human hearing). In a typical neuromodulation experiment, a pulse generator emits an electrical waveform, which is amplified and transferred to a transducer housing a piezoelectric element. This amplified electrical signal excites the element, causing the active face of the transducer to oscillate and produce ultrasound waves. The transducer is affixed to the head, often using a head strap, and with important technical developments (21) can be usable within a magnetic resonance imaging (MRI) scanner, to allow for MR-guided targeting. For example, as illustrated in Figure 1, this approach is accomplished in the scanner itself. Applying the transducer inside the bore provides several important and novel forms of data. First, using built-in fiducials, targeting occurs in real-time using a structural MR scan, with no interpolation of device elements or brain position. Furthermore, in-scanner application of LIFU allows causal estimation of observed deep-brain effects. Lastly, it also facilitates real-time safety assessments of brain structure and function.

### Mechanism of Ultrasound Neuromodulation

Like many other areas of brain stimulation, and more so because of its novelty, the exact mechanism underlying LIFU neuromodulation remains, yet, unknown (19, 22, 23). Research has demonstrated that ultrasound can interact with tissue to induce mechanical and thermal effects. One hypothesis posits that the low amount of acoustic radiation force alters permeability of mechanosensitive ion channels and voltage-gated calcium, sodium, and potassium channels in neuronal membranes (24). Another hypothesis postulates that vibration of extra- and intracellular environments produces mechanical changes in the plasma membrane tension or the lipid bilayer and modulates neuronal activities. Another possible mechanism centers on thermal effects. While an increase in tissue temperature could affect neuronal activity (and is the mechanism of action in irreversible ablative high intensity focused ultrasound), the temperature increase due to LIFU is often <0.1°C (22, 25) and effects are likely negligible (26).

### Sonication Parameters

Each transducer is composed of a piezoelectric element(s) that transforms electrical signal into ultrasound waves; the construction of this element also provides focality with a specific associated focal length (measured in mm) that defines a three-dimensional zone of maximum applied ultrasound, called “sonication.” A sonication protocol is defined by five parameters: (1) fundamental frequency, (2) pulse repetition frequency (PRF), (3) duty cycle, (4) sonication duration, and (5) intensity (23). The fundamental frequency refers to the number of oscillations over time and is inversely proportional to wavelength (and often referred to as a carrier wave). The PRF represents the rate at which acoustic pulses are delivered, and duty cycle is the proportion of each pulse filled with cycles of ultrasound at the fundamental frequency (or the ratio of “on time” to total time). The duration is quantified as the length of sonication, i.e., the total time from the onset of the first pulse to the termination of the final pulse. The spatial-peak temporal average (I_SPTA_) measures the average intensity during an entire sonication and the spatial-peak pulse average (I_SPPA_) measures the average intensity over a single pulse. The current US Food and Drug Administration safety guidelines for diagnostic ultrasound imaging devices suggest a maximum derated I_SPTA_ of 720 mW/cm^2^ and a maximum derated I_SPPA_ of 190 W/cm^2^ (27) to avoid heating and thermal damage. Derating (0.3 dB/cm·MHz; often indicated as a subscript, e.g., I_SPTA.3_) seeks to account for attenuation that occurs as the LIFU passes through tissue.

## Ultrasound Neuromodulation in Humans

In recent years, researchers have begun to use LIFU in humans, studying how different parameters can induce reversible physiological effects on the nervous system. Several foundational studies examined the effect of LIFU on neural activity using electroencephalography (EEG). The first study demonstrated that LIFU focally applied to the somatosensory cortex (Table 1) attenuated amplitudes of somatosensory-evoked potentials (SEPs) and enhanced sensory discrimination (28). The neuromodulatory effects of LIFU on SEPs were further investigated by leveraging its ability to target deep structures such as the thalamus. Sonication of the ventro-posterior lateral nucleus of the thalamus (Table 1) attenuated amplitudes of SEPs, and worsened the ability to perform difficult tactile threshold judgments (29).

**Table 1:** Human studies utilizing low intensity focused ultrasound neuromodulation.

**Study**	**Transducer (manufacturer)**	**Fundamental frequency**	**Sonication parameters**	**Brain target**	**Outcomes**
Legon et al. (28)	Single, focused (Blatek)	500 kHz	PRF: 1 kHz; DC: 36%; SD: 0.5 s; np = 500; I_SPPA_: 23.87 W/cm^2^	Primary somatosensory cortex	Improved sensory discrimination, SEP amplitude attenuation as assessed by EEG (*n* = 12 healthy participants)
Monti et al. (30)	Single, focused (Brainsonix)	650 kHz	PRF: 100 Hz; DC: 5%; 10 sonications, each lasting 30 s; I_SPTA.3_: 720 mW/cm^2^	Thalamus	Case study (*n* = 1): Recovery from severe brain injury post sonication
Legon et al. (29)	Single, focused (Ultran)	500 kHz	PRF: 1 kHz; DC: 36%; 300 sonications every 4 s; I_SPPA_: 7.03 W/cm^2^	Sensory thalamic nucleus	SEP suppression as assessed by EEG, Worsening tactile discrimination task (*n* = 40 healthy participants)
Legon et al. (31)	Single, focused (Ultran)	500 kHz	PRF: 1 kHz; DC: 36%; SD: 0.5 s; np = 500; I_SPPA_: 17.12 W/cm^2^	Primary motor cortex	MEP inhibition (*n* = 50 healthy participants)
Badran et al. (32)	Single, focused (Brainsonix)	650 kHz	PRF: 10 Hz; DC: 5%; 10 sonications, each lasting 30 s; I_SPTA.3_: 719 mW/cm^2^	Anterior thalamus	Thermal pain sensitivity attenuation (*n* = 19 healthy participants)
Yu et al. (33)	Single, focused (Blatek)	500 kHz	PRF: 300 Hz and 3 kHz; SD = 0.5 s; I_SPTA.3_: 702.58 mW/cm^2^	Primary motor cortex	Enhanced movement-related cortical potential as assessed by EEG (*n* = 15 healthy participants)
Cain et al. (34)	Single, focused (Brainsonix)	650 kHz	PRF: 100 Hz; DC: 5%; 10 sonications, each lasting 30 s; I_SPTA.3_: 719.73 mW/cm^2^	Left central thalamus	First-in-man clinical trial (*n* = 3): significant increases in behavioral responsiveness (*n* = 2/3); responsiveness to command, functional communication

In addition to SEP modulation, recent studies have examined LIFU on the motor cortex. Using a simultaneous ultrasound and TMS paradigm, LIFU to the motor cortex (Table 1) reduced the amplitude of TMS-evoked motor evoked potentials and attenuated intracortical facilitation (31). Conversely, a study using concurrent LIFU-EEG (Table 1), found evidence that LIFU enhanced the movement-related cortical potential (33). A novel theta burst patterned LIFU protocol was also recently introduced that produced a consistent increase in motor cortical excitability (35).

Badran et al. demonstrated that LIFU may be able to modulate pain sensitivity via thalamic sonication (32). Researchers administered MRI-guided LIFU targeting the right anterior thalamus in 19 healthy volunteers (Table 1) and observed that two 10-min sessions produced an antinociceptive effect on pain thresholds compared to sham (32). A first-in-human repetitive, pulsed LIFU platform targeting the hippocampus has also recently been developed and shown to be safe (36).

Presently there are very few reports of LIFU in patients with neurological or psychiatric disorders (Table 1). The first study of clinical application of LIFU was part of a first-in-human case report testing the feasibility, safety, and initial efficacy of thalamic LIFU in a 25-year-old patient with a severe traumatic brain injury and disorder of consciousness (30). Using MRI-guided LIFU, they observed improved alertness, language comprehension, response to commands, and reliable communication (30) following thalamic sonication. In a subsequent unblinded study, three patients with chronic disorder of consciousness received two sessions of MRI-guided LIFU to the left thalamus. Two of the three patients had improved behavioral responsiveness, and one regressed after 3 months (37).

Recently, investigators have investigated whether ultrasound may modulate mood and worry. Several studies have used transcranial ultrasound, a less focal ultrasound technique, to modulate activity in regions associated with mood and anxiety symptoms. In one double-blind pilot study, researchers applied continuous ultrasound over the posterior frontal cortex in 31 healthy participants and observed significant mood improvements at 10- and 40-min post-sonication (38). Another study showed that modulating the right inferior frontal gyrus (rIFG) with transcranial ultrasound could induce positive mood effects (39). In this study, researchers sonicated rIFG in a sample of 51 healthy volunteers and observed that one 30 s exposure of 500 kHz rIFG ultrasound could induce positive mood effects lasting up to 30 min (39).

Similarly, in a double-blind pilot study, researchers investigated the effect of right fronto-temporal cortex non-focal transcranial ultrasound on mood and worry in depressed patients (40). In this study, researchers sonicated the right fronto-temporal cortex in 24 patients with mild to moderate depression as part of a five-session protocol. Ultrasound was administered continuously and without pulsation. Patients received 30 s of sonication for five sessions occurring within 7 days, with sonication sessions separated by at least 1 day. Worry and positive mood scores improved after active group vs. sham stimulation (40).

Taken together, the above work represents initial evidence of the feasibility of ultrasound neuromodulation in humans. Non-focal transcranial ultrasound methods point to this technique's ability to alter mood states and set the stage for more focal LIFU experiments. This work also demonstrates that LIFU can target both cortical and deeper brain structures with lasting neuromodulatory effects and high spatial precision.

## Challenges and Limitations

### Safety

Establishing that LIFU can be safely applied to the brain is paramount in enabling this technique to become a viable research and potential clinical tool. The above studies provide preliminary evidence of safety, as no serious adverse events or brain injury were described. Most of these human studies provide safety data in the form of neurological examinations and/or structural MRI before and after sonication. Comprehensive reviews also suggest a favorable human safety profile (19, 26, 41, 42). A recent report (41) described qualitative safety and side effect assessments in 65 participants who previously completed one or more LIFU experiments. No participant, including those undergoing repeated LIFU, reported a serious adverse event. Mild to moderate effects, including neck pain, attention difficulties, muscle twitches, anxiety, and sleepiness were reported by 11% of participants and perceived as “possibly” or “probably” LIFU-related. In contrast, little information has been generated about histological changes in human brain tissue after ultrasound at or exceeding FDA intensity guidelines. As of this writing there is a single example, using tissue from cadavers or resected during neurosurgery. Investigators sonicated tissue at varying intensities and observed no histological changes at intensities up to 11,800 mW/cm^2^ I_SPTA.3_ (43), which substantially exceeds FDA guidelines. The safety of LIFU is echoed in animal work. Rodent, large animal, and primate studies have experimentally used a range of ultrasound frequencies (220 kHz−1.9 MHz) to produce effective neuromodulation without neurologic injury [reviewed in Fomenko et al. (23)]. One study in sheep reported that exposure to prolonged sonication incurred cerebral microhemorrhages (44), though this has been the only reported study to date reporting any injury across species.

While prior reports provide evidence for human safety, the majority of studies were conducted in healthy populations. To our knowledge, only three studies have examined safety in clinical patients, and early findings suggest relatively safety in those with brain injury (30, 37) or depression (40). We are currently evaluating safety as part of a series of first-in-human studies (U01 MH123427) utilizing radiological, psychiatric, and neuropsychological assessments in clinical patient populations, as clinical populations often have medical comorbidities (e.g., hypertension, smoking, etc.) associated with more fragile vasculature susceptible to acoustic injury.

### Dosing

Low intensity focused ultrasound dose–response curves for desired neurophysiological effects are not established. The parameter space is very large, as multiple parameters can be varied (e.g., fundamental frequency, pulse repetition frequency, duty cycle, sonication duration, and intensity) and the impact of altering parameters awaits systematic examination. Effects of repeated LIFU administration are also in need of evaluation. Furthermore, because LIFU administers low amounts of energy, it is likely that the brain state or context during sonication (e.g., rest vs. task) may affect the dose–response. The ideal way to combine sonication and manipulation of the neurocognitive state remains unclear (45) and may be of particular import in the application of low intensity modalities (46).

### Targeting and Delivery

The effect of variable skull anatomy across individuals is another important issue in LIFU research. Skull density and composition, particularly presence of trabecular (porous) bone between the cortical bone inner and outer tables, affect ultrasound conduction, as this porous nature leads to scattering. There are clear examples of this when using high intensity focused ultrasound (47, 48), however, the impact of the skull remains an unknown quantity for LIFU. To date, LIFU has often been applied over the temporal bone (i.e., with its cortical bone composition) to mitigate skull effects (29, 30). One immediate concern is the ability to focally deliver ultrasound when there is substantial variability in skull transmission. To address this issue, modeling (e.g., k-wave) approaches are being developed to determine how to accommodate individual skull variability (19, 49).

### Mechanisms of Action and Study Designs

A potential confound with respect to LIFU's ability to modulate deep brain structures is that it may activate intermediate regions like auditory cortex (50, 51). Several studies found that ultrasound-induced activities were eliminated or reduced upon auditory nerve transection or removal of cochlear fluids. These observations motivate careful attention to experimental designs rather than confounding effects.

Whether LIFU settings are more likely to excite, inhibit, or disrupt neural activity remains an important area of inquiry. To this effect, studies have tested whether PRF (52) or duty cycle (53) might determine the directionality of neuromodulation, with conflicting results. More in-depth studies on parameters and protocols are required to understand underlying mechanism(s) of LIFU neuromodulation.

Reflecting the very early nature of the field, there remains no quantifiable metric of LIFU-related physiological effects, with prior work in clinically relevant studies either relying upon behavioral output (30, 32) or indirect measures of perfusion (34). Given this limitation, future studies should include biological measures to provide insight into observed effects. Furthermore, challenges remain to ensure that investigators report parameters that can be easily compared to each other; this harmonization is currently an active area of effort with focused ultrasound experts across the globe (i.e., ITRUSST, https://itrusst.github.io/).

## Conclusions

Low intensity focused ultrasound holds appeal for its unique combination of non-invasiveness, likely safety, precision, and broad range of possible targets. Recent studies provide initial evidence of the safety and feasibility. These advances set the stage for pioneering studies to test causal hypotheses in neuropsychiatric research, utilizing focal, and reversible suppression of core brain regions involved in psychiatric illnesses. A virtually infinite parameter space and brain targets remain to be studied, and further prospective studies are needed to determine dose–response effects on neural activity. Yet, for those parametric studies to be successful, research is urgently needed (and currently ongoing) on safety, particularly in patient populations, as well as inquiry into targeting, acoustic confounds, and mechanisms of action. While the field's understanding of neural mechanisms underlying LIFU remain at a very early stage of study, it appears that the clinically relevant LIFU studies are consistently suppressive. Of note, we explicitly do not use the word inhibitory, as this conveys a degree of mechanistic characterization that is not yet supported by the extant literature.

Caveats aside, this technology has tremendous potential for clinical applications. Focal, non-invasive, deep-brain stimulation is currently impossible with available technologies. If the promise of LIFU-induced neuromodulation is realized, it may become not only a powerful tool to test causal relationships between brain activity and function, and transform clinical therapeutics.

## Author Contributions

AA wrote the original draft. All authors reviewed, edited, and approved the final version of the manuscript.

## Funding

This work was supported in part by the National Institutes of Health grants U01MH123427 and P20 GM130452, and the US Department of Veterans Affairs RR&D Center for Neurorestoration and Neurotechnology (I50 RX002864) and a CSR&D Career Development Award (IK2 CX001824). The contents do not represent the views of the National Institutes of Health, U.S. Department of Veterans Affairs, or the United States Government.

## Conflict of Interest

The authors declare that the research was conducted in the absence of any commercial or financial relationships that could be construed as a potential conflict of interest.

## Publisher's Note

All claims expressed in this article are solely those of the authors and do not necessarily represent those of their affiliated organizations, or those of the publisher, the editors and the reviewers. Any product that may be evaluated in this article, or claim that may be made by its manufacturer, is not guaranteed or endorsed by the publisher.

## Abbreviations

SEP: somatosensory evoked potential
MEP: motor evoked potential
PRF: pulse repetition frequency
DC: duty cycle
I_SPPA_: intensity spatial peak pulse average
I_SPTA.3_: derated intensity spatial peak temporal average
SD: sonication duration
np: number of pulses.
